# An RNA-seq time series of the medaka pituitary gland during sexual maturation

**DOI:** 10.1038/s41597-023-01967-w

**Published:** 2023-01-31

**Authors:** Eirill Ager-Wick, Gersende Maugars, Kristine von Krogh, Romain Fontaine, Finn-Arne Weltzien, Christiaan Henkel

**Affiliations:** 1 Physiology Unit, Faculty of Veterinary Medicine, Norwegian University of Life Sciences, Ås, Norway; 2 Present Address: Stress Environnementaux et BIOsurveillance des milieux aquatiques UMR-I 02 SEBIO, Université Le Havre Normandie, Le Havre, France

**Keywords:** Reproductive biology, Neurophysiology, Developmental biology, Gene expression analysis, RNA sequencing

## Abstract

Directing both organismal homeostasis and physiological adaptation, the pituitary is a key endocrine gland in all vertebrates. One of its major tasks is to coordinate sexual maturation through the production and release of hormones stimulating gonad development. In order to study its developmental dynamics in the model fish medaka (*Oryzias latipes*), we sampled both the pituitary and the ovaries of 68 female fish. Of these, 55 spanned the entire course of sexual maturation from prepubertal juveniles to spawning adults. An additional 13 showed either considerably faster or slower growth and development than the majority of fish. We used histological examination of the ovaries to determine a histological maturation stage, and analyzed the pituitary glands using RNA-seq optimized for low input. Taken together, these data reveal the timing of hormone production priorities, and form a comprehensive resource for the study of their regulation.

## Background & Summary

All animals seek to grow, reproduce, and maintain homeostasis – but all at the right time. Several endocrine glands coordinate the present physiological needs of the organism and communicate these to different organs and tissues. The pituitary is a key endocrine gland common to all vertebrates, involved in the regulation of many important physiological processes, which it modulates by releasing several peptide hormones into the bloodstream. By integrating signals derived from the brain, as well as feedback signals from downstream peripheral organs, the pituitary holds the focal position in several conserved endocrine regulatory systems, including the brain-pituitary-gonadal^1,2^ (BPG), brain-pituitary-thyroid^2^ (BPT) and brain-pituitary-adrenal/interrenal^3^ (BPA/BPI) axes.

Over the lifespan of an animal, the pituitary gland exhibits a high degree of developmental plasticity, allowing the proliferation or reduction of specific cell types to meet changing demands^4^. Distinct cell types are responsible for the production and secretion of at least eight different peptide hormones^5,6^. Sexual maturation, for instance, is controlled by pituitary gonadotrope cells producing follicle-stimulating and luteinizing hormones (Fsh and Lh, respectively). These cells are themselves under the control of several brain-derived factors (e.g. the hypothalamic gonadotropin-releasing hormone, Gnrh) and subject to feedback through sex steroids produced by the gonads and other organs^7^. Understanding pituitary mechanisms is, therefore, a central goal of endocrinological research, as it provides a key handle on the experimental management of an animal’s physiology. In the case of teleost fish, this knowledge has important applications in aquaculture, ecology, and conservation.

One of the most important experimental perspectives on pituitary mechanisms is the transcriptome. Supported by high-throughput sequencing methods, it allows the sensitive inventory of a tissue’s investment in specific functions. As an early example of the potential of transcriptomics, the last major new peptide hormone discovered in the pituitary – the teleost-specific somatolactin – was identified based on its cDNA sequence alone^8^.

To study the development of pituitary mechanisms over the course of sexual maturation, we here describe a transcriptomics dataset for the pituitary gland of the model fish medaka, *Oryzias latipes*. As medaka are very small fish (9.5–29 mm in this study), we used a transgenic line^9^ that expresses green fluorescent protein in Lh gonadotropes, facilitating the dissection of pituitary glands. We sampled pituitaries from individual female fish aged 32 to 375 days post-fertilization, and profiled these using a low-input Illumina RNA-seq protocol.

For each fish, sexual maturity was assessed using histological examination of the ovaries. Medaka is a daily spawner, and its follicles mature in an asynchronous manner – the ovaries will contain follicles at several levels of maturity. Therefore, we took the largest observed follicle size as a measure of overall gonadal maturity.

The time series confirms that the teleost pituitary goes through major changes during development and sexual maturation. All established pituitary peptide hormones are expressed at very high levels, but not all of them at all stages of development. Either *pomc* (encoding the hormone precursor pro-opiomelanocortin, Ensembl identifier ENSORLG00000025908), *prl* (prolactin-1, ENSORLG00000016928), or *gh1* (growth hormone, ENSORLG00000019556) is the most abundant transcript in nearly all samples; their expression changes relatively little during development. By contrast, both *lhb* (Lh β subunit, ENSORLG00000003553) and *fshb* (Fsh β subunit, ENSORLG00000029237) clearly increase during maturation.

There exists considerable variation in growth, maturation, and gene expression between individuals, with some fish showing marked deviations in developmental timing. In all but the most extreme cases, these animals do not show unusual hormone gene expression profiles. In general, the intra-individual variation does offer a window into the cell type composition of the pituitary gland. The time series thereby complements and extends our earlier efforts using single-cell RNA-seq, which was based on larger pools of only sexually mature animals^6^.

## Methods

### Experimental design

In order to capture major changes in the pituitary during sexual maturation, we designed a time series experiment in which we bred Japanese medaka (*Oryzias latipes*) siblings (offspring from three couples) under controlled conditions. We sampled the pituitaries and ovaries of female fish after 32–178 days post-fertilization (dpf). The youngest fish were juveniles, and the smallest from which the pituitary gland can be reliably dissected; fish were fully sexually mature well before the final age. In addition, we sampled three older fish (249–277 dpf) from the same facility and the three female parents (375 dpf). For all fish, we collected gonads to estimate sexual maturity; a fin clip to confirm female sex by genotyping; and the pituitary gland for RNA-seq profiling. The animal experiments performed for this study were approved and performed in the model fish facility at the Faculty of Veterinary Medicine of the Norwegian University of Life Sciences (registered at the Norwegian Food Safety Authorities under FOTS VSID 2859, 170 NMBU Veterinærhøgskolen).

### Fish husbandry and growth experiment

We used medaka of the d-rR genetic background that express green fluorescent protein under the control of the *lhb* promotor^9^, facilitating pituitary dissection even in small animals. We sampled eggs from a tank containing three females and three males. After hatching in petri dishes, fish from a single egg collection date were transferred to 1-liter tanks in a re-circulation system. After one month, fish were transferred to 3-liter tanks containing exactly 10 fish of the same age. Fish were kept at 28 °C on a 14/10 h light/dark cycle, and were fed three times a day with a mix of dry food and *Artemia*.

### Fish sampling

We sampled 118 female fish from our time series experiment (aged 32–178 days after egg collection), as well as the three female parents (375 days) and three mature females of intermediate age (249–277 days), housed in the same facility. The fish were sacrificed between 09.00–11.00 h in the morning by hypothermic shock (immersion in ice water) to minimize distress^10^, followed by severing the spinal cord, and immediate dissection of the pituitary. Pituitaries were carefully dissected using fine forceps and extra care was taken to prevent cross-contamination between samples by carefully cleaning all equipment with ethanol after dissecting each fish, and also before sampling the pituitary. Each pituitary gland was put in a 0.5 ml Eppendorf tube containing 10 µl ice-cold medaka-adjusted^11^ PBS (pH 7.75 and mOsm 290 mOsm) and 5 ceramic beads of 1.4 mm (MP Biomedials), directly followed by the addition of 300 µl Trizol Reagent (Invitrogen) to each tube. The sample was vortexed at high speed for 1 minute and snap frozen in liquid nitrogen. Samples were stored at −80 °C until RNA isolation.

Subsequently, we collected ovaries, transferred them to small glass bottles containing 4% glutaraldehyde (Merck Millipore) 0.1 M phosphate-buffered solution (pH 7.2), and left them to incubate overnight at 4 °C. The samples were then stored in 70% EtOH at 4 °C until histological processing. Whenever possible, ovaries were dissected out and examined macroscopically to obtain a preliminary maturity estimate. Fin clips were collected for sex genotyping.

### Sex genotyping

Genomic DNA was isolated from fin clips by incubation in 25 μl alkaline lysis buffer (25 mM NaOH, 0.2 mM EDTA) for 5 minutes at 95 °C and subsequent neutralization using 25 μl 40 mM Tris-HCl (pH 8.0). Genotypic sex was determined by genomic PCR for the presence or absence of the *dmy* gene (primers 5′-CCGGGTGCCCAAGTGCTCCCGCTG and 5′-GATCGTCCCTCCACAGAGAAGAGA) and analysis by agarose gel electrophoresis^12,13^. All fish in the dataset were confirmed female.

### Transcriptome sequencing

We selected 68 samples for sequencing. Of 105 fish for which pituitary and gonads sampling, as well as genotyping were successful, 84 fit a general growth and maturation trend. Of these, we randomly selected 47 fish, as well as the two youngest and the six oldest (parents and independent intermediate age fish), for a total of 55 fish. 21 fish were possible growth curve outliers. Of these, we included the three exhibiting faster than average growth, and 10 of 18 showing slow growth and maturation (at random), for a total of 13 outlier samples.

RNA was isolated using a standard Trizol protocol, with minor modifications. Samples were thawed and vortexed for 10 seconds with 120 µl chloroform, incubated at room temperature for 3 minutes, and centrifuged at 14000 g for 15 minutes at 4 °C. Glycoblue (17.5 µg/ml, Invitrogen) was added to each tube to enhance precipitation and visualize the pellet. The aqueous phase (ca. 160 µl) was transferred to a new tube and RNA was precipitated in 160 µl 100% isopropanol by incubation at −20 °C for 1 hour, followed by centrifugation at 14000 g for 15 minutes at 4 °C. The pellet was then washed in 300 µl 75% ice-cold ethanol and the sample centrifuged at 10000 g for 10 minutes at 4 °C. The supernatant was removed and the RNA pellet briefly dried (approximately 5 minutes) at room temperature. The pellet was dissolved in 10 µl of RNase-free water. To avoid unnecessary freeze-thawing of the samples, aliquots of 2 µl of each sample were made for Bioanalyzer analysis. Samples were stored at −80 °C until use.

In order to ascertain that only samples with high quality RNA were used for sequencing, we investigated RNA concentration and integrity of all samples using the Bioanalyzer Eucaryote total RNA pico kit. The Bioanalyzer software did not always assign an RNA integrity (RIN) score, as RNA concentrations were often very low. In 11 cases, we therefore validated the electrophoresis profile by visual inspection. For the other 57 samples, the RIN was high (mean 9.2, median 9.5), with the lowest value of 6.8 assigned to one of the smallest fish (sample 109, 10 mm).

RNA was shipped on dry ice for library preparation and sequencing at Future Genomics Technologies (Leiden, The Netherlands). cDNA was generated and amplified using the Smart-SEQ HT Kit for low RNA input according to the manufacturer’s recommendations (Takara Bio), which was used for generating sequencing libraries with the Nextera XT kit. Libraries were sequenced at 151 nt paired-end on an Illumina NovaSeq 6000 system, yielding 9.5–16.2 million reads per sample.

### Histological analysis

The fixated tissue was dehydrated in a series of increasing concentrations of EtOH (70–100%), each step lasting at least 30 minutes. The last step (100%) was repeated three times and then replaced with approximately 5 ml of preparation solution (100 ml Technovit 7100 with 1 g of Hardener I, Heraeus Kulzer) and kept at slow shaking at room temperature overnight. After infiltration, tissue samples were embedded in cold Histoform S (Heraeus Kulzer) with approximately 1 ml preparation solution and 50 µl Hardener II (Heraeus Kulzer) and incubated at 37 °C. Cured samples were mounted on Histoblocs using Technovit 3040 (both from Heraeus Kulzer). The gonads were sectioned using a Leica Biosystems RM2245 microtome. Sagittal sections (3 µm) were made from the periphery until the middle of the gonad and collected on microscope slides every 30–90 µm, depending on the gross maturity of the gonad. Dried sections were stained with Toluidine Blue O (Sigma-Aldrich) and mounted with Coverquick 4000 (VWR International) prior to microscopy analysis.

Sectioned ovarian tissue was inspected using a Nikon Eclipse Ci light microscope, and images were acquired using a Lumenera Infinity 2–5 C camera. To measure the size of the oocytes, make captions, and setting bars on the pictures, Lumenera Infinity Analyze software (version 6.2) was used. Raw images were light and/or sharpness adjusted and cut to desired size. The developmental stage (following ref. ^14^) of the tissue was determined by the most advanced ovarian follicle present (see Table 1).

**Table 1:** Time series fish per gonadal maturation stage.

Stage	Description (stage follicle size in µm)	Observed follicle size (µm)	Nr. of fish	Nr. confirmed	Age (dpf)	Length (mm)
n.a.	No estimate	n.a.	2	0	45–48	14.0–14.5
II	Early previtellogenic (60–90)	70–90	10	6	32–64	9.5–15.5
III	Late previtellogenic (91–120)	95–120	9	9	37–59	11.0–14.5
IV	Late previtellogenic (121–150)	130–150	4	4	44–66	11.0–16.0
V	Early vitellogenic (151–250)	160–200	7	6	52–99	14.5–16.5
VI	Early vitellogenic (251–400)	260–300	2	2	74–79	15.0–17.0
VII	Late vitellogenic (401–500)	430–480	3	2	87–99	17.5–18.5
VIII	Late vitellogenic (501–800)	600–760	4	4	87–111	17.5–19.0
IX–X	Mature (801–1200)	≥1000	13	13*	96–375	18.0–29.0
II–IX	Fast-growing outliers	150–1000	3	2	66–74	19.0–20.0
II–VI	Slow-growing outliers	105–260	10	7	66–111	10.0–15.0

Gonadal maturation stage is based on initial macroscopic observations of the tissue, and was confirmed by histological examination (based on follicle size^14^) for 46 fish. * For 9 fish, sexual maturity was confirmed by the presence of externally visible eggs (approximate size 1200 µm), obviating the need for histological examination.

### Sequencing data analysis

All FASTQ data were aligned against the medaka HdrR reference genome (Ensembl version 94) using STAR^15^ (version 2.7.6a). Alignments were inspected or processed using Samtools^16^ (version 1.10) and quantified using htseq-count^17^ (version 0.11.2) using the *intersection-nonempty* setting. The resulting data were analyzed in R (version 4.0.3) using the edgeR^18^ (version 3.32.0) and cqn^19^ (version 1.36.0) packages.

We focused on genes that show medium to high expression in the pituitary. As a selection criterion, we took the appearance in any sample in the top 1000 protein-coding genes by expression level. Over all samples, this resulted in a set of 2462 medium to high expression genes, which suggests samples differ considerably by expression profile. Differential expression between maturity cohorts, and along age, length, or principal components of the dataset were calculated using edgeR using the GLM functionality. Initial *p*-values were corrected using the Benjamini-Hochberg procedure to control the false discovery rate at 5%.

## Data Records

**Fig. 1:**
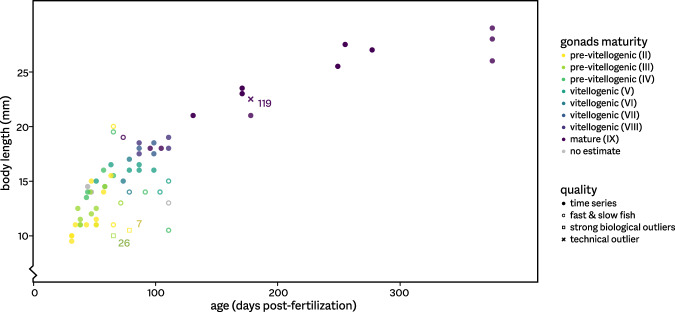
Growth and maturation in female medaka. The 67 fish analyzed in this study range from young, small and sexually immature (pre-vitellogenic gonads) to old, large and sexually fully mature. Gonad maturation status was assessed using histology for most samples, following the stages described by Iwamatsu *et al*. (ref. ^14^); for a few fish, we only obtained macroscopic estimates, and for two no estimates. We did not perform histological staging for the gonads of most of the mature, spawning fish. Closed circles indicate the 54 samples taken as part of the main time series, 11 samples flagged during the growth experiment as especially fast- or slow-growing are indicated by open circles. Two further slow-growing fish (7 and 26) are indicated by open squares – these biological outliers also show a relatively strongly deviating transcriptomic profile (see Figs. 3,4). One technical outlier sample (119), not included in any further analyses, is marked by a cross.

The dataset contains single pituitary RNA-seq profiles for 68 individual fish: 55 for which growth correlates well with age, and 13 outliers identified during sampling (Fig. 1 & Table 1). One RNA-seq profile of the main time series was discarded after sequencing (Fig. 1., see Technical Validation section). Raw sequencing files are available from the NCBI Gene Expression Omnibus^20^ and Sequence Read Archive^21^ (see Table 2 for accession number ranges). Sample information, processed RNA-seq data, and R code for RNA-seq data processing are available at NMBU Open Research Data (DataverseNO)^22^. Included in the processed RNA-seq data are raw read counts (per gene, per sample) and normalized data for the medium-high expressed genes (normalized using both quantile and scaling methods, see the Technical Validation section). For the 54 quantile-normalized samples of the main time series, differential expression results are available for a number of contrasts.

**Table 2:** Complete contents of Gene Expression Omnibus and Sequence Reads Archive records.

Description	Sample nr. range	GEO accessions	SRA accessions	Analyzed in study
65 female time series	1–121	GSM5421298– GSM5421317, GSM5421336, GSM5421338– GSM5421381	SRX11365370– SRX11365389, SRX11365402– SRX11365421, SRX11365428, SRX11365430–SRX11365453	This study
3 female adults	122–124	GSM5421318– GSM5421320	SRX11365390– SRX11365392	This study; ref. ^6^
12 male juveniles	125–136	GSM5421321– GSM5421332	SRX11365393– SRX11365401, SRX11365422– SRX11365424	Not analyzed
4 male adults	137–140	GSM5421333– GSM5421335, GSM5421337	SRX11365425– SRX11365427, SRX11365429	ref. ^6^

All entries are available in the main GEO^20^ and SRA^21^ accessions.

**Fig. 2:**
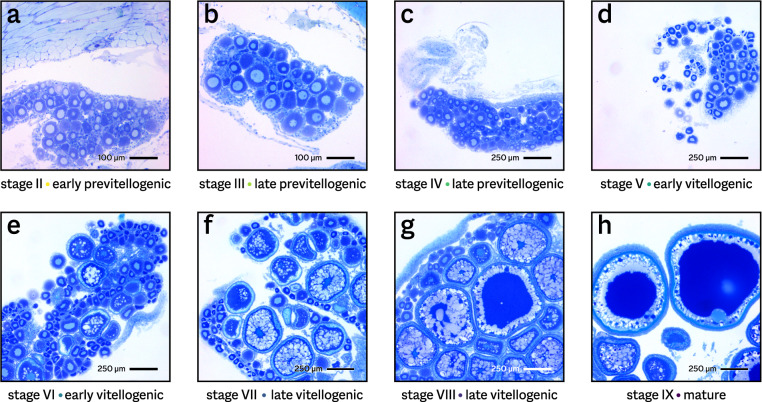
Histological confirmation of gonadal maturation. Shown are example images of the eight stages. The size of the largest observed follicle determines the maturation status (see Table 1). Bars correspond to 100 µm (panels a and b, 100 × magnification) or 250 µm (panels c–h, 40 × magnification). Samples shown here are (by fish number) (**a**) 109; (**b**) 14; (**c**) 24; (**d**) 5; (**e**) 4; (**f**) 18; (**g**) 63; and (**h**) 115.

For all but two of these animals, we obtained an initial estimate of sexual maturation stage by macroscopic observation of the gonads. For 55 of those, we were able to confirm and refine the staging by histological examination (Fig. 2 & Table 1). These microscopy images are also included in the NMBU Open Research Data record^22^.

## Technical Validation

### Reproducibility and outlier detection

**Fig. 3:**
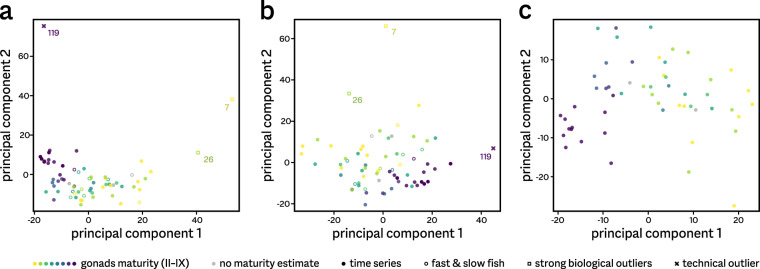
Outlier detection and sample reproducibility. (**a**,**b**) Principal component analysis (PCA) of all pituitary transcriptome profiles identified three samples as potential outliers. Mature fish 119 (cross) is strongly divergent at the transcriptomic level only, and was removed from the analyses. Samples 7 and 26 are also transcriptomic outliers, however they represent unusual biological states (slow growth, see Fig. 1) and were therefore retained in the dataset. Shown are the first two components for PCA on log-transformed expression values of medium-high expressed genes using quantile (**a**) or scaling (**b**) sample normalization. (**c**) PCA after outlier (119) removal and quantile re-normalization, using medium-high expression genes and main time series samples only. Principal component 1 shows a clear parallel with maturation (colour scale as in Fig. 1).

We initially assessed the consistency of the (normalized) RNA-seq dataset using a set of 2686 genes expressed in the top 1000 in any sample. A principal component analysis (PCA) using these genes identified a single sample (119, from a mature fish) as a strong technical outlier (Fig. 3a,b). Detailed examination revealed an apparent compound profile consisting of a typical pituitary expression signature combined with very strong expression of genes not expressed in any other sample, suggesting either sample contamination with non-pituitary tissue or atypical gland composition. We therefore did not include this single sample in further analyses. Removing sample 119 from the dataset reduced the ‘top 1000’ of medium-highly expressed genes to 2462 genes.

**Fig. 4:**
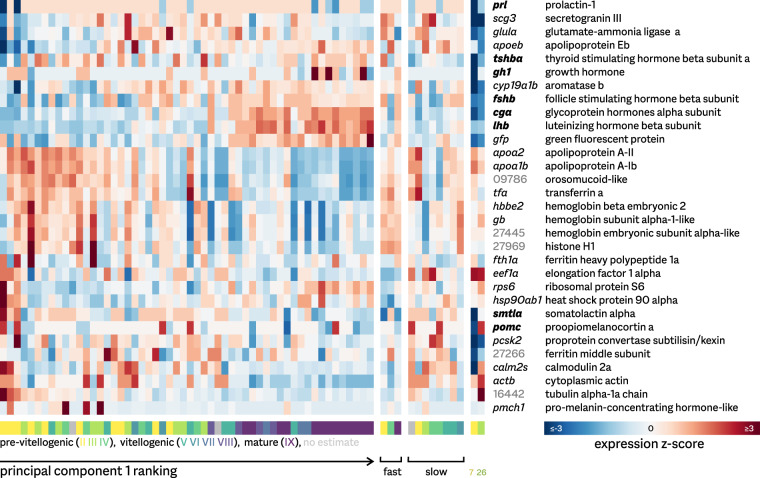
Expression changes during development and maturation. Heatmap of the variation in expression of the most highly expressed genes in the medaka pituitary, defined as ranking in the top 10 in any of the 54 samples of the main time series. Also included are the fast and slow maturation groups, as well as the two slowly growing outlier samples. Variation is shown as standard deviation around the mean (z-score) for each gene. Genes are hierarchically clustered by expression pattern. Genes in bold encode the major pituitary protein hormones; numbers (in grey) are shorthand for Ensembl identifiers of unnamed genes (e.g. 09786 refers to gene ENSORLG00000009786). Time series samples are ordered by the first principal component of the RNA-seq profiles (see Fig. 3).

Several other samples also show deviating tendencies; however, these do not show highly atypical expression patterns. In particular, samples 7 and 26 are relatively strong outliers (Fig. 3a,b). These originate from fish that were identified as biological outliers during sampling, and we therefore retained them in the full dataset. Interestingly, their expression profiles show a clear de-prioritization of nearly all peptide hormone-encoding genes, but not of the other genes highly expressed in the pituitary (Fig. 4).

**Fig. 5:**
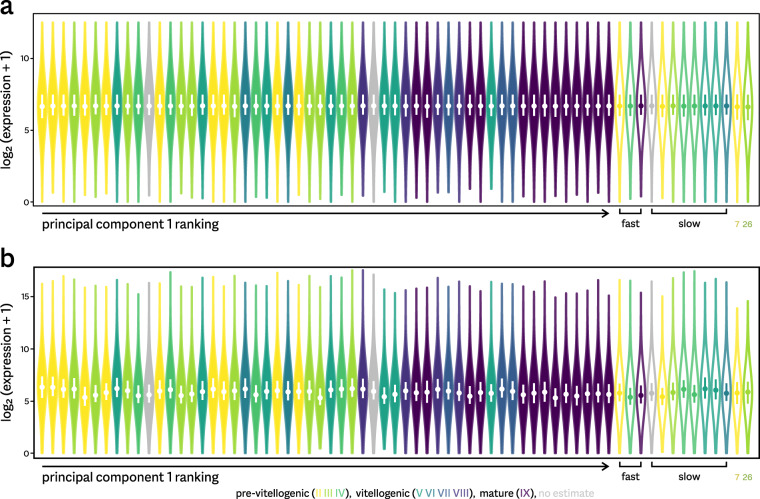
Alternative normalization strategies. Shown are expression levels per sample, normalized using either a quantile strategy (**a**) or TMM scaling (**b**). Violin plots show the distributions of the levels for medium-high expression genes, with median values (circles) and the lower to upper quartile range (bars) indicated. After quantile normalization, expression profiles are comparable between samples; scaling normalization does not succeed in making samples comparable.

In a reduced dataset, we only included 54 samples derived from fish that followed a normal course of development (see Fig. 1). When subjected to PCA, no clear outliers are present anymore (Fig. 3c). The first principal component (explaining 22.2% of variance within the data) appears to correlate well with development, and was used to unambiguously order samples for Figs. 4 & 5.

### Biological quality control

As medaka pituitary glands are very small and challenging to sample, it is important to ascertain that the resulting transcriptomic profiles are consistent with pituitary gene expression profiles. Figure 4 presents a heatmap of the most abundant transcripts over all samples. All genes encoding pituitary protein hormones are included in this set, indicative of a good representation of the pituitary gland in general. In addition, variations in expression patterns are globally correlated with development.

To investigate whether expression trends can be linked to development and maturation in general, we performed differential expression analyses along the time series. Of the 2462 medium-high expression genes, 66.2% and 62.6% change significantly with age and body length, respectively (5% false discovery rate). When grouped by developmental stage, 48.0% of genes are differentially expressed between pre-vitellogenic (combined stages II–IV) and mature (IX) fish, 15.9% between pre-vitellogenic and vitellogenic (V–VIII), and 27.3% between vitellogenic and mature. These sets are composed of comparable proportions of up- and downregulated genes, as expected for well-normalized data.

### RNA-seq normalization

As our data were both amplified from low input material and derived from developmentally heterogeneous samples, they do not fit the assumptions for normalization by scaling commonly applied to RNA-seq (e.g. minimal changes in gene expression between samples). We therefore normalized counts for the medium-high expression genes between samples using quantile normalization as previously described^23^. This essentially yields rank-like expression values, enabling comparisons (including differential expression analysis) between samples.

In order to validate this choice, we also normalized the data by scaling, using either the Trimmed Mean of M-values^24^ (TMM) or DESeq2-like^25^ estimates of scaling factors. Both gave very similar results (Pearson correlation *r* = 0.99 between the sets of normalization factors). Figure 5 illustrates the distribution of gene expression values obtained using quantile and TMM normalization. The first equalizes the distributions (Fig. 5a), whereas the latter fails to do so (Fig. 5b). In particular, using scaling normalization, the pituitary samples from sexually mature fish appear to show lower overall expression, which would have a major effect on any downstream analysis. For example, differential expression analysis would flag the majority of genes as downregulated, not counterbalanced by any upregulation. Although reduced transcriptional activity could be a genuine biological phenomenon, one would not expect to see this represented in sequencing data after RNA extraction, cDNA amplification, and library preparation. A more plausible explanation is the (expected) impossibility of deriving a single sample scaling factor to equalize such data.

Our available dataset includes data processed by both methods. We recommend using the quantile-normalized values for most purposes, including all comparisons between samples. For comparisons within each sample, these data may be less suitable, as the procedure dampens large variation in expression levels (see the difference in y-axis scale in Fig. 5a,b).

## Data Availability

R code used in the analyses is included in the NMBU Open Research Data record^22^.

## References

[1] Dufour S (2020). Origin and evolution of the neuroendocrine control of reproduction in vertebrates, with special focus on genome and gene duplications. Physiol. Rev..

[2] Sower SA, Freamat M, Kavanaugh SA (2009). The origins of the vertebrate hypothalamic–pituitary–gonadal (HPG) and hypothalamic–pituitary–thyroid (HPT) endocrine systems: New insights from lampreys. Gen. Comp. Endocrinol..

[3] Denver RJ (2009). Structural and functional evolution of vertebrate neuroendocrine stress systems. Ann. N. Y. Acad. Sci..

[4] Fontaine R (2020). Gonadotrope plasticity at cellular, population and structural levels: A comparison between fishes and mammals. Gen. Comp. Endocrinol..

[5] Weltzien F-A, Andersson E, Andersen Ø, Shalchian-Tabrizi K, Norberg B (2004). The brain–pituitary–gonad axis in male teleosts, with special emphasis on flatfish (Pleuronectiformes). Comp. Biochem. Physiol. A. Mol. Integr. Physiol..

[6] Siddique K, Ager-Wick E, Fontaine R, Weltzien F-A, Henkel CV (2021). Characterization of hormone-producing cell types in the teleost pituitary gland using single-cell RNA-seq. Sci. Data.

[7] Fontaine, R., Royan, M.R., Von Krogh, K., Weltzien, F.-A. & Baker, D.M. Direct and indirect effects of sex steroids on gonadotrope cell plasticity in the teleost fish pituitary. *Front. Endocrinol*. **11** (2020). 10.3389/fendo.2020.605068 PMC7750530 33365013

[8] Ono M (1990). cDNA cloning of somatolactin, a pituitary protein related to growth hormone and prolactin. Proc. Natl. Acad. Sci..

[9] Hildahl J (2012). Developmental tracing of luteinizing hormone β-subunit gene expression using green fluorescent protein transgenic medaka (*Oryzias latipes*) reveals a putative novel developmental function. Dev. Dyn..

[10] Köhler A (2017). Report of workshop on euthanasia for zebrafish – a matter of welfare and science. Zebrafish.

[11] Ager-Wick, E. *et al*. Preparation of a high-quality primary cell culture from fish pituitaries. *JoVE J. Vis. Exp*. e58159 10.3791/58159 (2018). 10.3791/58159 PMC6231918 30222142

[12] Shinomiya A, Otake H, Togashi K, Hamaguchi S, Sakaizumi M (2004). Field survey of sex-reversals in the medaka, *Oryzias latipes*: genotypic sexing of wild populations. Zoolog. Sci..

[13] Ansai S, Kinoshita M (2014). Targeted mutagenesis using CRISPR/Cas system in medaka. Biol. Open.

[14] Iwamatsu T, Ohta T, Oshima E, Sakai N (1988). Oogenesis in the medaka *Oryzias latipes* – stages of oocyte development. Zoolog. Sci..

[15] Dobin A (2013). STAR: ultrafast universal RNA-seq aligner. Bioinformatics.

[16] Li H (2009). The Sequence Alignment/Map format and SAMtools. Bioinformatics.

[17] Anders S, Pyl PT, Huber W (2015). HTSeq—a Python framework to work with high-throughput sequencing data. Bioinformatics.

[18] Robinson MD, McCarthy DJ, Smyth GK (2010). edgeR: a Bioconductor package for differential expression analysis of digital gene expression data. Bioinformatics.

[19] Hansen KD, Irizarry RA, Wu Z (2012). Removing technical variability in RNA-seq data using conditional quantile normalization. Biostat. Oxf. Engl..

[20] Ager-Wick E (2021). NCBI Gene Expression Omnibus.

[21] (2021). NCBI Sequence Read Archive.

[22] Ager-Wick E, Maugars G, Von Krogh K, Weltzien F-A, Henkel C (2021). DataverseNO.

[23] Ager-Wick E, Henkel CV, Haug TM, Weltzien F-A (2014). Using normalization to resolve RNA-Seq biases caused by amplification from minimal input. Physiol. Genomics.

[24] Robinson MD, Oshlack A (2010). A scaling normalization method for differential expression analysis of RNA-seq data. Genome Biol..

[25] Anders S, Huber W (2010). Differential expression analysis for sequence count data. Genome Biol..

